# An inducible model of chronic hyperglycemia

**DOI:** 10.1242/dmm.050215

**Published:** 2023-08-04

**Authors:** Tori R. Tucker, Courtney A. Knitter, Deena M. Khoury, Sheida Eshghi, Sophia Tran, Abigail V. Sharrock, Travis J. Wiles, David F. Ackerley, Jeff S. Mumm, Michael J. Parsons

**Affiliations:** ^1^ Department of Developmental and Cell Biology, University of California, Irvine, Natural Sciences II, Irvine, CA 92697, USA; ^2^ Department of Molecular Biology and Biochemistry, University of California, Irvine, Natural Sciences II, Irvine, CA 92697, USA; ^3^ School of Biological Sciences, Victoria University of Wellington, Wellington 6012, New Zealand; ^4^ Department of Ophthalmology, The Wilmer Eye Institute, Johns Hopkins University, Baltimore, MD, USA

**Keywords:** Cell-specific ablation, Nitroreductase, Metronidazole, Chronic hyperglycemia, Diabetes

## Abstract

Transgene driven expression of *Escherichia coli* nitroreductase (NTR1.0) renders animal cells susceptible to the antibiotic metronidazole (MTZ). Many NTR1.0/MTZ ablation tools have been reported in zebrafish, which have significantly impacted regeneration studies. However, NTR1.0-based tools are not appropriate for modeling chronic cell loss as prolonged application of the required MTZ dose (10 mM) is deleterious to zebrafish health. We established that this dose corresponds to the median lethal dose (LD_50_) of MTZ in larval and adult zebrafish and that it induced intestinal pathology. NTR2.0 is a more active nitroreductase engineered from *Vibrio vulnificus* NfsB that requires substantially less MTZ to induce cell ablation. Here, we report on the generation of two new NTR2.0-based zebrafish lines in which acute β-cell ablation can be achieved without MTZ-associated intestinal pathology. For the first time, we were able to sustain β-cell loss and maintain elevated glucose levels (chronic hyperglycemia) in larvae and adults. Adult fish showed significant weight loss, consistent with the induction of a diabetic state, indicating that this paradigm will allow the modeling of diabetes and associated pathologies.

## Teaser

**Summary:** Taking advantage of a new nitroreductase with higher activity, we developed transgenic zebrafish lines that can be used to induce chronic hyperglycemia and can be used to model a diabetic state.

## INTRODUCTION

Both type 1 diabetes (T1D) and type 2 diabetes (T2D) are characterized by chronic hyperglycemia. Autoimmunity in T1D results in the loss of insulin-producing β cells of the pancreas. T2D is caused by the inability to produce enough insulin or the development of insulin resistance. Although diabetes is mainly categorized as T1D or T2D, the disease is more complex. For instance, maturity-onset diabetes of the young (MODY) is caused by inheritance of monogenetic mutations (Fajans and Bell, 2011). Regardless of etiology, prolonged hyperglycemia in diabetic patients leads to damage to various tissues, including the eyes, kidneys, blood vessels and nerves (Pourghasem et al., 2015; Shukla and Tripathy, 2022; Singh et al., 2014). Various animal systems have been used to model hyperglycemia (Acharjee et al., 2013; Bakker and de la Garza, 2022; Kottaisamy et al., 2021; Wolf et al., 2014; Zettler et al., 2020). The non-obese diabetic (NOD) mouse has a genetic predisposition for autoimmune diabetes that mimics features of human T1D (Chen et al., 2020). Streptozotocin (STZ) injection in rodents is an often-used method to ablate β cells and model diabetes (Furman, 2015). STZ is an alkylating agent that enters cells through the Glut2 (also known as SCL2A2) receptor and damages DNA, leading to cell death. β cells highly express Glut2, making them more susceptible to STZ.

More recently, zebrafish have emerged as a model to study diabetes. As in mammals, the zebrafish pancreas has both an endocrine (in the form of β-cell-containing ‘islets’) and an exocrine compartment (Tiso et al., 2009). Through the first 5 days of development, the pancreas contains a single principal islet, which is maintained into adulthood. Subsequently, secondary islets form that are scattered throughout exocrine tissue (Parsons et al., 2009). Unlike other animal models, zebrafish are highly regenerative (Gemberling et al., 2013), making them an excellent model to study β-cell neogenesis (Kinkel and Prince, 2009; Prince et al., 2017; Tarifeño-Saldivia et al., 2017; Kimmel and Meyer, 2016).

When STZ is injected into adult fish, it induces β-cell death and a corresponding increase in blood glucose levels (Moss et al., 2009; Olsen et al., 2012, 2010; Sarras et al., 2015). However, use of STZ in zebrafish is complicated as the high doses necessary for β-cell ablation are also associated with damage to tissues outside the pancreas and lethality (Moss et al., 2009). Genetic methods to study hyperglycemia in the zebrafish include mutations in *pdx1*. MODY4 in humans is caused by haploinsufficiency in PDX1. Zebrafish lacking Pdx1 (*pdx1*^−/−^ mutants) have reduced numbers of β cells as larvae, and the few fish that survive to adulthood display elevated blood glucose levels (Kimmel et al., 2015) and retinopathy (Wiggenhauser et al., 2020) However, it is hard to separate symptoms caused by chronic diabetes from developmental defects. Transgenesis can provide temporal control, allowing the induction of chronic hyperglycemia after embryonic/juvenile stages. Several different transgenic approaches have been used to ablate β cells in zebrafish (Ibrahim et al., 2020; Li et al., 2009; Moro et al., 2009), but the most widely used method is the nitroreductase (NTR)/metronidazole (MTZ) method (Pisharath et al., 2007; Curado et al., 2007; Ghaye et al., 2015; Yu et al., 2023; Singh et al., 2022; Ye et al., 2015; Carril Pardo et al., 2022; Kim et al., 2023).

Transgenically expressed NTR converts the antibiotic MTZ into a cell-restricted cytotoxin, enabling inducible, targeted cell ablation. The original NTR was cloned from the *Escherichia coli nfsB* gene and is referred to here as NTR1.0. The insulin promoter was used to express both NTR1.0 and a fluorescent marker in β cells to create a line facilitating the destruction of β cells and loss of insulin production. Hyperglycemia was induced in adult transgenic fish by injection of a single dose (30 mmol/l) of MTZ dissolved in PBS at a dose of 0.25 g/kg body weight. Subsequent β-cell regeneration led to a return to normal glucose levels (euglycemia) after 2 weeks (Delaspre et al., 2015). Although the NTR1.0/MTZ system has worked well in studying β-cell regeneration, it has not been a suitable method to induce chronic hyperglycemia, or model diabetes, as maintenance of the high dose of MTZ is detrimental to the health of the fish (Pisharath et al., 2007). Here, we reveal that even acute high-concentration MTZ (10 mM) treatments lead to lethality and inflammation-associated aberrant gut morphology in surviving zebrafish.

Recently, Sharrock et al. (2022) engineered an alternative NTR (termed NTR2.0), derived from the *Vibrio vulnificus nfsB* gene (*nfsB_Vv F70A/F108Y*), which has higher MTZ conversion activity, enabling effective cell ablation at ∼100-fold lower doses of MTZ. We used the insulin promoter (Pisharath et al., 2007) to drive NTR2.0 expression in transgenic zebrafish and demonstrate that complete β-cell ablation can be achieved with dramatically lower doses of MTZ, which are better tolerated by fish of all ages. The higher ablation efficacy of NTR2.0 allowed us to adopt immersion as the method of MTZ delivery in both larvae and adults and to carry out chronic hyperglycemia trials. We report on protocols to keep larvae hyperglycemic for 10 days and adults hyperglycemic for 16 days. Further, we show that chronic hyperglycemia in adults leads to severe weight loss, consistent with the pathology of diabetes.

## RESULTS

### NTR2.0 requires less MTZ to ablate β cells

**Fig. 1.**
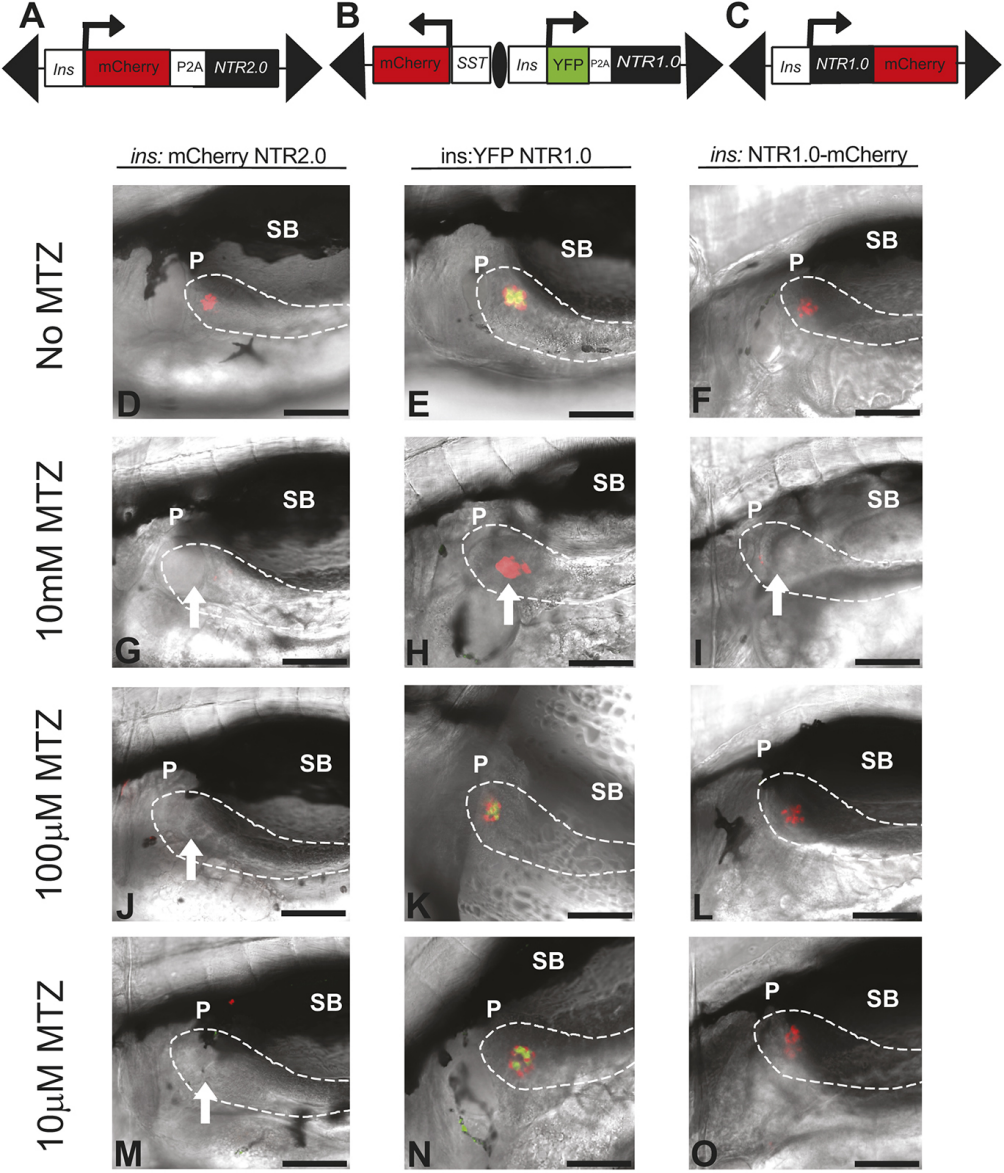
**Use of NTR2.0 improves efficacy in ablating β cells.** (A-C) Schematic of NTR2.0 (A) and NTR1.0-based transgenes (B,C), with positions of Tol2 arms (black triangles), HS4 insulator (black oval) and promoters (arrows) indicated. (D-O) Larvae were incubated from 3 to 5 dpf (48 h) in MTZ at various concentrations (as indicated) and imaged. White arrows point to the position of ablated β cells. Results from *ins:mCherry NTR2.0* larvae (D,G,J,M) compared to both *ins:YFP NTR1.0^lmc01^* (E,H,K,N) and *ins:NTR1.0-mCherry^jh4^* (F,I,L,O) are shown. Only larvae expressing NTR2.0 achieve a complete loss of β cells when treated with 100 µM MTZ (J). Dashed white lines outline the pancreas. All images were taken at 40× (scale bars: 100 µM) with anterior to the left. dpf, days post fertilization; MTZ, metronidazole; NTR, nitroreductase; P, pancreas; SB, swim bladder.

To improve β-cell ablation in zebrafish, we cloned the insulin promoter upstream of a cassette (*GAP-tagYFP-P2A-NTR2.0*) to express both membrane-tagged YFP (GAP-tagYFP) and NTR2.0 (Hall et al., 2022; Sharrock et al., 2022) (Fig. S1A). The whole transgene was flanked by Tol2 transposable arms, and Tol2-mediated transgenesis was used to insert the construct into the genome (Kawakami, 2004). Following microinjection and the derivation of multiple F1 lines, we observed that expression of this construct led to fewer labeled β cells than expected and that those few labeled cells displayed blebbing – evidence of cell stress (Fig. S1B,C). We suspected that this might be due to the membrane-tagged reporter (Huang et al., 2000; Liu et al., 1999). Accordingly, we generated two new transgenic lines that lacked a membrane tag and were named Tg(*ins:mCherry-P2A-nfsB_Vv F70A/F108Y*)*^ir2018^* (abbreviated to *ins:mCherry NTR2.0^ir2018^*) (Fig. 1A) and Tg(*ins:YFP-P2A-nfsB_Vv F70A/F108Y*)*^ir2050^* (abbreviated to *ins:YFP NTR2.0^ir2050^*) (Fig. S2A). After outcrossing the lines for two generations, transgenic fish from both lines transmitted their transgenes to 50% of their progeny, indicating that both transgenes inserted at a single locus. PCR analysis demonstrated that the transgenes entered the genome via Tol2-mediated transgenesis (Fig. S3). The significance of using transgenic lines that only have a single site of insertion is that all related hemizygous transgenic fish will contain the same number of insertions. Ensuring an identical number of insertions reduces experimental variation and facilitates the maintenance of a functional line of fish. Next, we functionally analyzed our new lines for β-cell ablation efficacy.

A range of MTZ concentrations (0, 10 µM, 100 µM and 10 mM) was tested on *ins:mCherry NTR2.0^ir2018^* larvae from 3 to 5 days post fertilization (dpf). Results were compared to those from similarly treated control larvae lacking NTR (*ins:dsRed^m1018^)* (Fig. S4A) and larvae from pre-existing NTR1.0 models, namely *ins:NTR1.0-mCherry^jh4^* (Pisharath et al., 2007) and *ins:PhiYFP-nfsB;sst2:TagRFP^lmc01^* (abbreviated to *ins:YFP NTR1.0^lmc01^*) (Delaspre et al., 2015; Walker et al., 2012) (Fig. 1). *ins:dsRed^m1018^* larvae (*n*=30) were incubated in 10 mM MTZ for 48 h without any effects on β-cell numbers, verifying that β-cell ablation is MTZ and NTR dependent (Fig. S4B). As previously reported, only 10 mM MTZ was sufficient to completely ablate β cells in *ins:YFP NTR1.0^lmc01^* (50/50) and *ins:NTR1.0-mCherry^jh4^* (50/50) larvae (Fig. 1H,I). In contrast to in NTR1.0-expressing fish, lower doses of MTZ ablated β cells in *ins:mCherry NTR2.0^ir2018^* larvae; 100 µM MTZ completely ablated β cells in all (50/50) larvae tested (Fig. 1J) and in 70% (35/50) of larvae treated with 10 µM MTZ (Fig. 1M). As identical results were also obtained for *ins:YFP NTR2.0^ir2050^* larvae (*n*=30) (Fig. S2C,D), we adopted 100 µM MTZ as a working dose in all our subsequent larval studies.

### Testing for transgene silencing

**Fig. 2.**
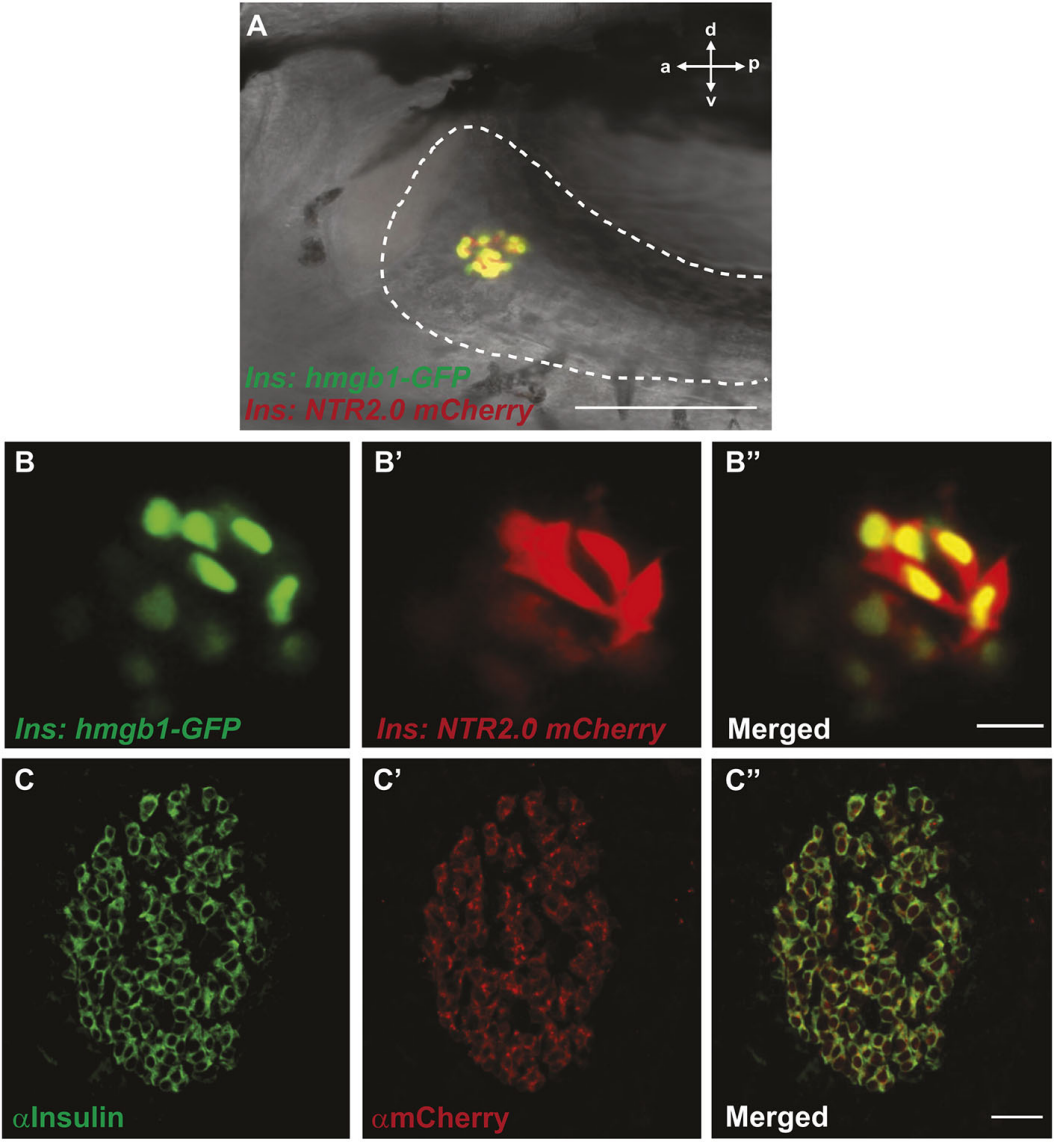
**Testing for transgene silencing.** (A-B″) Live confocal images of 5 dpf larvae carrying two transgenes *ins:hmgb1-eGFP* (labels β-cell nuclei green) and *ins:mCherry NTR2.0* (designed for β-cell expression of NTR2.0 and mCherry)*.* Both transgenes mark islet cells in the head of the pancreas (20×, scale bar: 100 µm) (A). Close up of green nuclei (B) and red cytoplasm (B′) and co-expression in β cells (B″), indicating correct β-cell-specific expression of NTR2.0 (40×, scale bar: 10 µm). (C-C″) Confocal images of sections from *ins:mCherry NTR2.0* fish with immunofluorescent detection of insulin (to detect β cells, green; C) and mCherry (red, reports on NTR2.0 co-expression; C′); complete colocalization can be seen throughout the islet (C″) (40×, scale bar: 10 µm). a, anterior; d, dorsal; p, posterior; v, ventral.

Epigenetic silencing can complicate the creation of transgenic tools. β cells not expressing NTR would escape MTZ-induced cell death but would perhaps prevent hyperglycemia and complicate interpretation of ablation experiments. NTR2.0 from our transgenes was co-expressed with fluorescent proteins to report on expression. We crossed our new lines to well-characterized β-cell reporter lines and imaged the double transgenic progeny for colocalization of fluorescent proteins by confocal microscopy. *ins:mCherry NTR2.0^ir2018^*, *ins:hmgb1-GFP^jh10^* (Wang et al., 2015) larvae showed complete colocalization of mCherry (red) and nuclear GFP (green) in all β cells, indicating correct larval expression of NTR2.0 (Fig. 2A-B″). Similarly, *ins:YFP NTR2.0^ir2050^*, *ins:dsRed^m1018^* (Shin et al., 2008) larvae showed complete colocalization of YFP and dsRed (Fig. S2B-B″). To test for correct NTR2.0 expression in *ins:mCherry NTR2.0^ir2018^* adults, mCherry and endogenous insulin were detected by immunofluorescence (Fig. 2C-C″). Results demonstrated that all insulin-positive cells also contained mCherry, indicating sustained expression of NTR2.0 in adult β cells.

### Minimizing MTZ toxicity

**Fig. 3.**
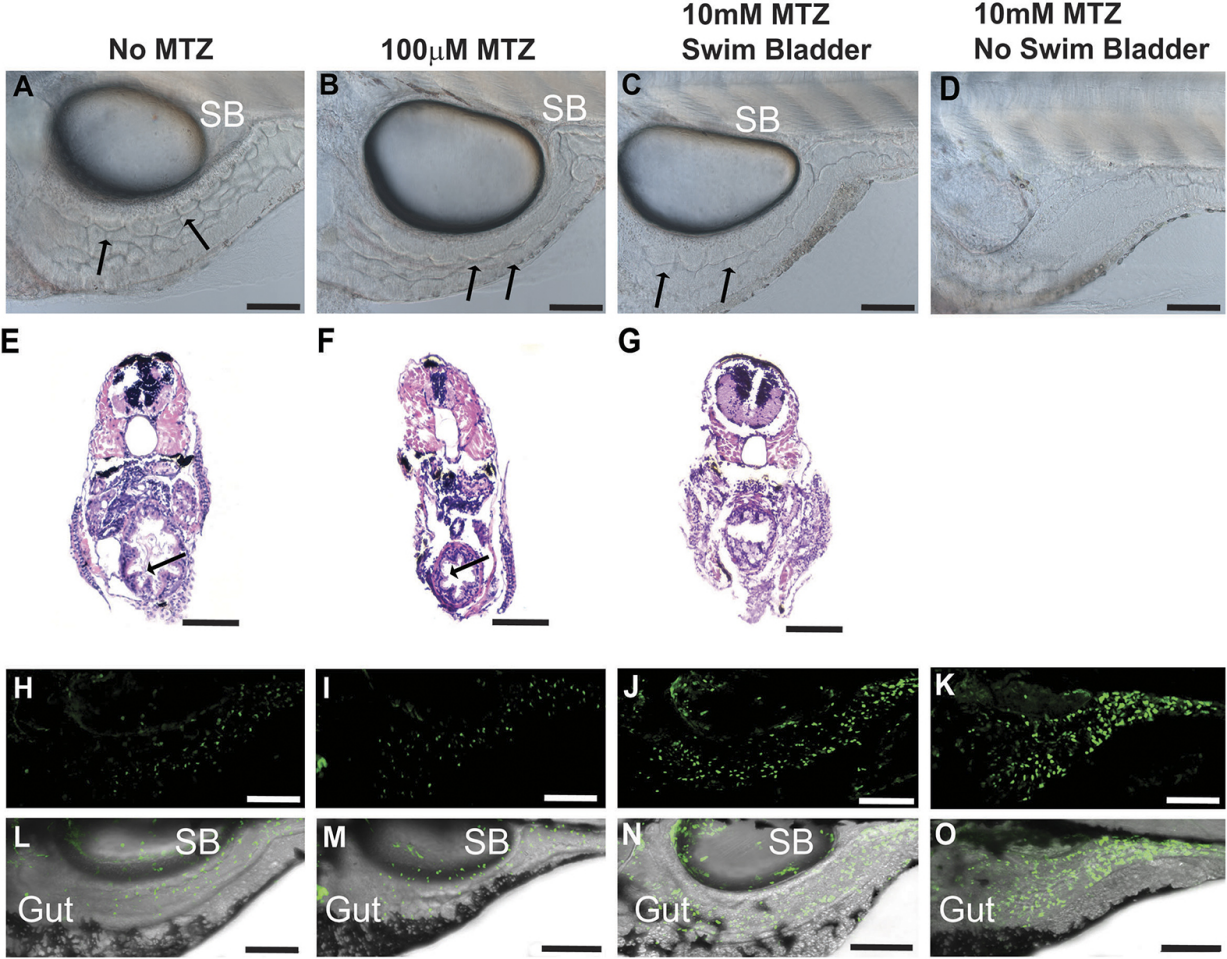
**Deleterious effects of 10 mM MTZ on larval zebrafish.** (A-O) Images taken from 5 dpf control (0 mM MTZ) larvae (A,E,H,L) or larvae treated from 3 to 5 dpf with either 100 µM MTZ (B,F,I,M) or 10 mM (C,D,G,J,K,N,O). (A,B) Images from the trunk region of *casper* larvae, showing that control (A) and 100 µM MTZ-treated (B) larvae have discernible gut lumens (complete with normal intestinal folds) as well as inflated swim bladders. (C,D) Surviving larvae treated with 10 mM display less defined gut lumen with few intestinal folds; some larvae have inflated swim bladders (C) and others have no inflated swim bladder (D) (20×, scale bars: 100 µm). (E-G) Transverse sections through the trunk stained with H&E to observe intestinal morphology confirmed a lack of intestinal folds in 10 mM MTZ-treated larvae with an inflated swim bladder (G) (20×, scale bars: 100 µm). (H-O) Images from *8xNFκB:eGFP* 5dpf larvae indicate that 10 mM MTZ treatment leads to an increase in the number of cells undergoing NFκB signaling (J,K,N,O) (20×, scale bars: 100 µm). Black arrows point to intestinal folds. MTZ, metronidazole; SB, swim bladder.

For our transgenic lines to be suitable models of hyperglycemia, it is important that the amount of MTZ needed for complete β-cell ablation should not adversely affect fish health. To understand better the potential caveats of MTZ, we decided to investigate the potential toxic effects of MTZ on larval fish. In pilot experiments, we observed that 10 mM MTZ treatment from 3 to 5 dpf often led to failure to inflate the swim bladder, aberrant gut morphology and even lethality. Accordingly, we decided to do a larger experiment and document survival, gross morphology, gut histology and inflammation in larvae (without NTR transgenes) treated with a range of MTZ concentrations (0, 100 µM and 10 mM) from 3 to 5 dpf. To aid visualization of internal gross morphology, we used non-pigmented *casper* larvae (White et al., 2008). The failure to inflate the swim bladder by 5 dpf is an indicator of abnormal development and ill health (Driever et al., 1996) (Fig. 3A-D). Transverse paraffin sections were stained with Hematoxylin and Eosin (H&E) to visualize intestinal morphology (Fig. 3E-G), and inflammation was monitored by detection of GFP expression in an NFκB reporter line, *Tg*(*8xNFκB:eGFP)^ir20^*^19^ (Fig. 3H-O).

Larvae incubated without MTZ (*n*=50) or 100 µM MTZ (*n*=50) all survived and possessed inflated swim bladders (Fig. 3A,B). Five fish from each group were processed for histology; all these fish had normal gut morphology (Fig. 3E,F). Half the larvae immersed in 10 mM MTZ (25/50) died before 5 dpf; the surviving larvae were unhealthy, with aberrant digestive tracts as observed at the level of gross morphology (Fig. 3C,D). The survivors in this 10 mM MTZ group could be separated into two categories: (1) 13 larvae that inflated their swim bladders (Fig. 3C) and had abnormal gut histology that lacked the presence of intestinal folds (5/5 of the 13 fish in the group, Fig. 3G); and (2) 12 larvae that failed to inflate their swim bladders (Fig. 3D). Members of this latter group were also very fragile, which precluded tissue processing for histology (attempted on 5/12 fish in the group). To further characterize the intestinal phenotype, we used *8xNFκB:eGFP^ir2019^* larvae kept as negative controls (no MTZ, Fig. 3H,L), or treated with 100 µM MTZ (Fig. 3I,M) or 10 mM MTZ (Fig. 3J,K,N,O).

*8xNFκB:eGFP^ir2019^* transgenic fish treated without MTZ or with 100 µM MTZ were indistinguishable, with similar numbers of NFκB signaling cells around the digestive tract (Fig. 3H,I,L,M). This result led us to conclude that 100 µM MTZ had caused no inflammation in the intestinal region. Regardless of whether the MTZ-treated fish had inflated (Fig. 3J,N) or non-inflated (Fig. 3K,O) swim bladders, 10 mM MTZ led to an increase in the numbers of cells undergoing NFκB signaling, an observation consistent with digestive tract inflammation.

### MTZ-induced gut inflammation occurs in microbiota-depleted larval fish

**Fig. 4.**
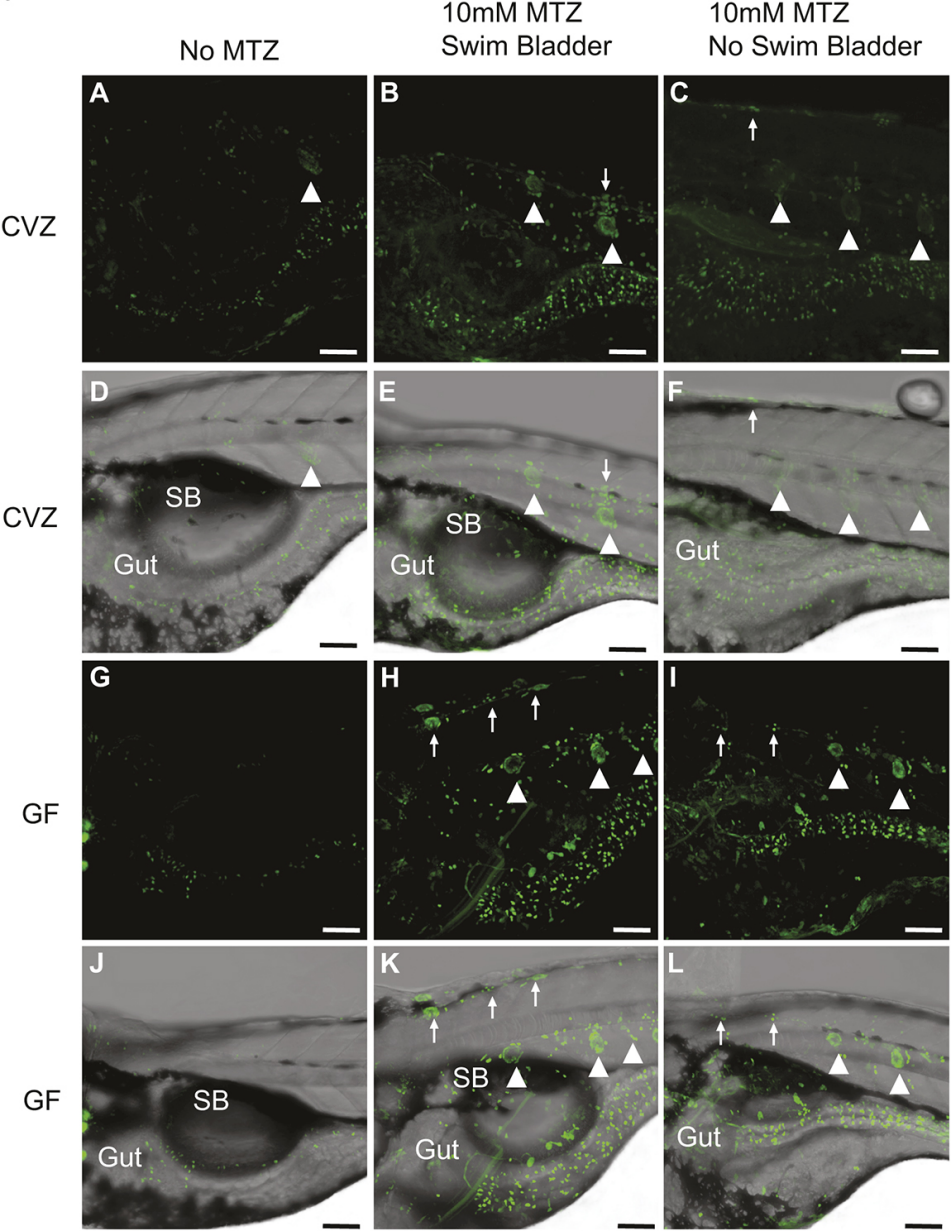
**10 mM MTZ has toxic effects on larval tissues independent of microbiota.** (A-L) Confocal images of CVZ (A-F) and GF (G-L) *8xNFκB:eGFP* 5 dpf larvae that were either kept as a negative control (no MTZ, A,D,G,J) or treated with 10 mM MTZ from 3 to 5 dpf (10 mM MTZ, B,C,E,F,H,I,K,L). MTZ-treated larvae either inflated or failed to inflate their swim bladders. Both CVZ and GF larvae showed the same response – an increase in the number of cells undergoing NFκB signaling in the gut, neuromasts (arrowheads) and skin (arrows) when treated with 10 mM MTZ. (A-C,G-I) 20× *z*-stacked fluorescent images. (D-F,J-L) Same *z*-stack imposed over brightfield image. Scale bars: 100 µM. CVZ, conventionalized; GF, germ free; MTZ, metronidazole; SB, swim bladder.

To test whether the apparent lethality and tissue damage was due to direct toxicity of MTZ or indirect alterations of resident microbiota mediated by MTZ antibiotic activity, we derived germ-free (GF) *8xNFκB:eGFP^ir2019^* larvae. Half the larvae were maintained in GF conditions and half were conventionalized (CVZ), a process that involves the reintroduction of conventional microbes following GF derivation (Melancon et al., 2017). In each group, the larvae were further divided, with one half treated with 10 mM MTZ from 3 to 5 dpf and one half kept as a negative control. CVZ and GF fish treated with 10 mM MTZ from 3 to 5 dpf produced the same response as seen previously in conventional ‘non-GF’ larvae (Fig. 3H-O). Again, about half of the 10 mM MTZ-treated fish failed to inflate their swim bladders (8/20 CVZ, Fig. 4F; 8/20 GF, Fig. 4L), and all treated CVZ fish (20/20, Fig. 4B,C) and all treated GF fish (20/20, Fig. 4H,I) showed an increase in the number of cells expressing GFP from the NFκB reporter. As tissue damage occurred after the depletion of micro-organisms, we concluded that 10 mM MTZ is directly toxic to the larval tissues we evaluated. Consistent with this conclusion, looking outside the digestive tract we observed increased numbers of cells undergoing NFκB signaling in extraintestinal tissues, namely the neuromast and skin (Fig. 4B,C,E,F,H,I,K,L). This increase was not seen in larvae not treated with MTZ (Fig. 4A,D,G,J).

### Acute β-cell ablation in adult fish

It was previously reported that 20 h immersion in 10 mM MTZ was sufficient to induce β-cell loss in *ins:NTR1.0-mCherry^jh4^* fish (Ghaye et al., 2015). To test the efficacy of this approach, we immersed 20 wild-type adult fish in 10 mM MTZ for 24 h. Half these fish died (*n*=10), and, during the treatment, all fish displayed both anorexia and lethargy. All surviving fish displayed some level of intestinal pathology (Fig. S5).

**Fig. 5.**
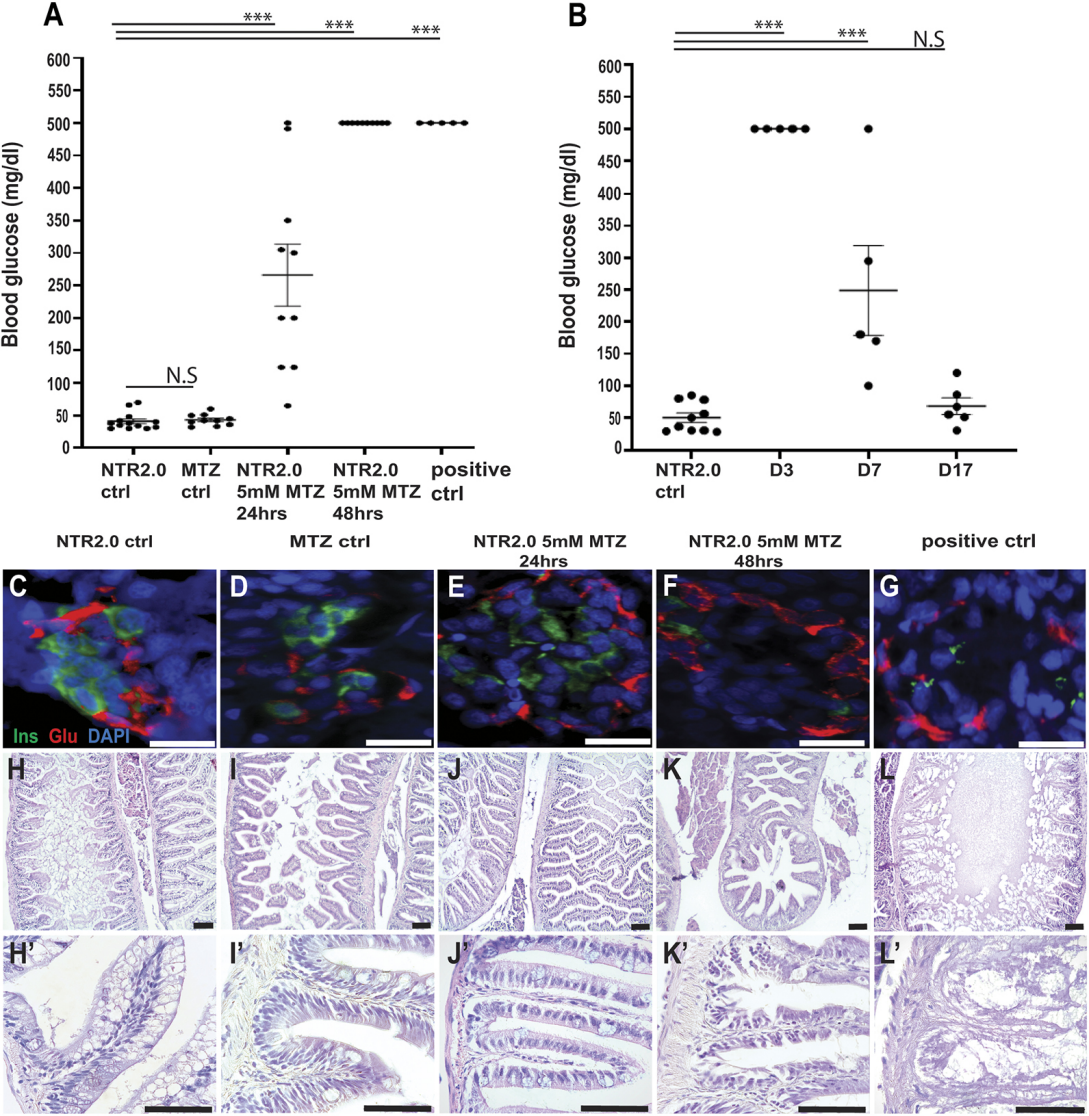
**Acute β-cell ablation in adults by MTZ immersion.** (A) FBG readings from five groups of adult fish: untreated *ins:mCherry NTR2.0^ir2018^* fish (NTR control), wild-type fish immersed in 5 mM MTZ for 24 h (MTZ control), *ins:mCherry NTR2.0^ir2018^* fish immersed for either 24 h or 48 h in 5 mM MTZ (NTR2.0 5 mM MTZ 24 h or 48 h), and *ins:mCherry NTR2.0^ir2018^* fish immersed in 10 mM MTZ for 24 h (positive control). Owing to the limitation of the blood glucose meter used, 500 mg/dl is the maximum FBG reading that can be captured. (B) FBG readings of NTR2.0 control and *ins:mCherry NTR2.0^ir2018^* fish. The NTR2.0 fish were treated with 5 mM MTZ for 48 h and were then allowed to recover for either 3, 7 or 17 days, after which FBG measurements were taken. N.S., not significant; ***P<0.05 (unpaired two-tailed Student's *t*-test). (C-G) Confocal images of paraffin sections through the pancreas after immunofluorescent detection for the islet markers insulin (green) and glucagon (red). Nuclei were counterstained with DAPI (blue) (40×, scale bars: 10 µm). (H,I,J,K,L) H&E histological analysis of intestines from fish in each treatment group (20×, scale bars: 100 µm). (H′,I′,J′,K′,L′) Magnification of same images at 60× (scale bars: 50 µm). ctrl, control; D, day; FBG, fasting blood glucose; MTZ, metronidazole; NTR, nitroreductase.

To test whether a lower dose of MTZ would be less toxic, we immersed *ins:mCherry NTR2.0^ir2018^* fish in 5 mM MTZ for either 24 h (NTR2.0 5 mM MTZ 24 h, *n*=10) or 48 h (NTR2.0 5 mM MTZ 48 h, *n*=10) and compared them to the following controls: untreated *ins:mCherry NTR2.0^ir2018^* fish (NTR2.0 control, *n*=13), wild-type fish immersed in 5 mM MTZ for 24 h (MTZ control, *n*=10) and *ins:mCherry NTR2.0^ir2018^* fish treated with the median lethal dose (LD_50_) of 10 mM MTZ (positive control, *n*=10) (Fig. 5). For all groups, we documented survival and general health. On completion of the trial, all fish had their fasting blood glucose (FBG) levels measured. It should be noted that the maximum FBG measurement that the FreeStyle lite glucose meter we used in this study is 500 mg/dl. The actual levels are likely to be higher (Delaspre et al., 2015). Five fish from each experimental and control group were also processed for histology and immunodetection of islet markers. Anti-glucagon antibodies were used to label α cells in the islet periphery, and anti-insulin antibodies were used to label β cells – the target for ablation. For each of the five fish sampled from each group, 15 islets were imaged by confocal microscopy.

All MTZ (10/10) and NTR2.0 controls (13/13) survived this experiment, had normal FBG levels (Fig. 5A) and had a healthy appearance. These fish also had normal intestinal epithelium (5/5, Fig. 5H,I) and complete islets containing β cells (5/5, Fig. 5C,D). NTR2.0 5 mM MTZ 24 h fish also survived, appeared healthy (10/10) and had normal gut morphology (5/5, Fig. 5J,J′). However, unlike the MTZ and NTR2.0 controls, these fish showed evidence of hyperglycemia, although with a high degree of variability (Fig. 5A). Consistent with FBG distribution in NTR2.0 5 mM MTZ 24 h fish, immunodetection of islet markers also demonstrated variability in β-cell ablation. Of the 75 islets examined (five fish, 15 islets from each fish), ten islets had normal proportions of β cells, 15 islets had no β cells, and 50 islets contained normal-appearing β cells along with immunoreactive cell debris consistent with ongoing, but incomplete, β-cell ablation (representative image in Fig. 5E).

All NTR2.0 5 mM MTZ 48 h fish survived and looked healthy (10/10). Five fish were processed for histology, and most of these fish showed normal intestinal morphology (4/5, Fig. 5K,K′); only one fish showed some limited pathology in the intestine (1/5, Fig. S5E). Consistent with complete β-cell ablation, very little insulin immunoreactivity was evident (5/5 fish, 75 islets in total) and was restricted to either cell debris or cells with fragmented 48 nuclei (indicative of apoptotic cells) (Fig. 5F). Of the positive control fish (*ins:mCherry NTR2.0^ir2018^* fish in 10 mM MTZ for 24 h, *n*=10), only half survived (5/10). All five of the surviving fish displayed hyperglycemia (Fig. 5A), β-cell loss (Fig. 5G) and an intestinal pathology whereby severely damaged areas (Fig. 5L) were interspersed within normal epithelium (Fig. S5C). In summary, immersion of *ins:mCherry NTR2.0^ir2018^* adult fish in 5 mM MTZ for 48 h leads to the same level of hyperglycemia and β-cell ablation as can be seen for treatment in 10 mM for 24 h, but without the associated gut pathology and lethality.

Our new model of acute adult β-cell ablation uses a different enzyme (NTR2.0) and a different drug delivery method (immersion in 5 mM MTZ for 48 h). The duration of exposure, and the rates of MTZ uptake, catalysis to active drug and cellular accumulation/retention are all likely to be different from those in our prior methodology whereby MTZ was injected (Delaspre et al., 2015). To test whether these changes altered the pattern of regeneration after acute β-cell ablation, we immersed *ins:mCherry NTR2.0^ir2018^* adult fish (*n*=16) in 5 mM MTZ for 48 h. Treated fish were sacrificed, and FBG levels were measured at 3, 7 and 17 days after removal from 5 mM MTZ – time points chosen to match our previous study (Delaspre et al., 2015). Mean (±s.d.) FBG readings were as follows: 3 days, ≥500 mg/dl (*n*=5); 7 days, 249±140.2 mg/dl (*n*=5); and 17 days, 68.1±28.6 mg/dl (*n*=6) (Fig. 5B). The results for day 17 were statistically comparable to those for samples from untreated NTR2.0 control fish (NTR2.0 control) – 50.2±22.0 mg/dl (*n*=10), demonstrating a return to euglycemia. This pattern of FBG recovery is identical to that in our previous study and led us to conclude that our new method of acute β-cell ablation via immersion of NTR2.0-expressing fish in MTZ had no significant effect on the dynamics of regeneration.

### Maintaining hyperglycemia during development

**Fig. 6.**
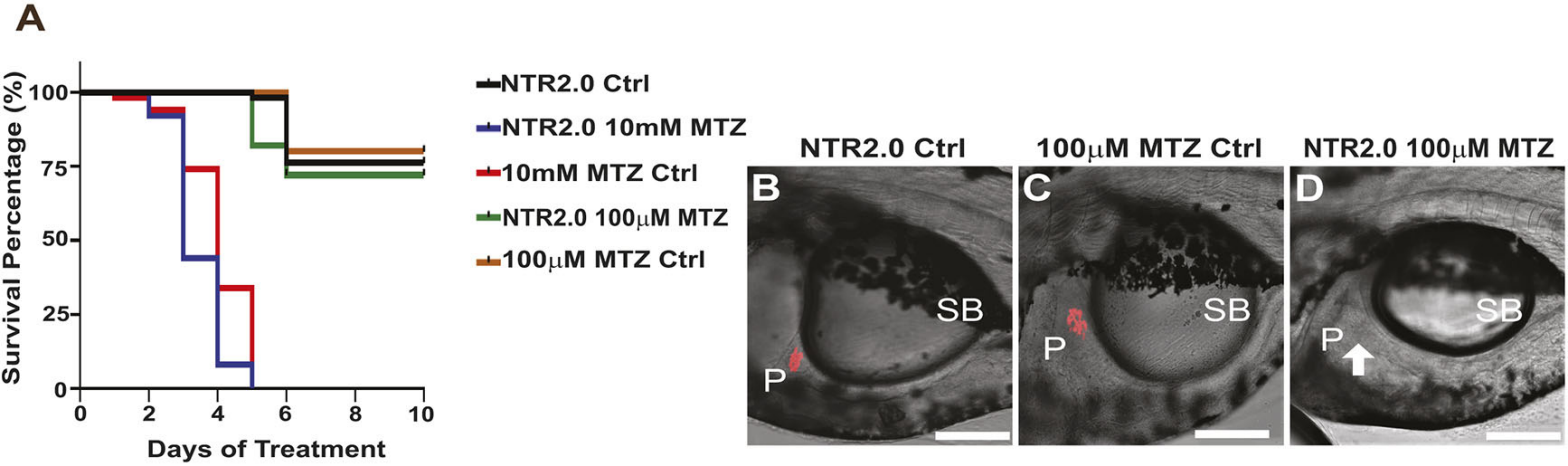
**Chronic hyperglycemia can be achieved in larvae using NTR2.0 fish.** (A) Survival curve: *ins:mCherry NTR2.0^ir2018^* larvae treated for 10 days (3 dpf to 13 dpf) with no MTZ (NTR2.0 Ctrl), 100 µM MTZ (NTR2.0 100 µM MTZ) or 10 mM MTZ (NTR2.0 10 mM MTZ). Other controls included *ins:dsRed^m1018^* larvae treated with 10 mM MTZ (10 mM MTZ Ctrl) and 100 µM MTZ (100 µM MTZ Ctrl). (B,C) Confocal images (each a single optical section) showing the presence of fluorescently labeled β cells in the trunk region of 13 dpf control larvae: NTR2.0 Ctrl (B) and 100 µM MTZ Ctrl (C). (D) β cells are not detected (white arrow) in NTR2.0 100 µM MTZ larvae, indicating that this treatment is sufficient to ablate β cells and prevent the regeneration new β cells. Scale bars: 100 µm. Anterior to the left, dorsal at the top. P, pancreas; SB, swim bladder.

Having established a significantly improved model of acute β-cell ablation, we next tested whether *ins:mCherry NTR2.0^ir2018^* fish could be used to model chronic hyperglycemia during larval stages. *ins:mCherry NTR2.0^ir2018^* (*n*=30) and control *ins:dsRed^m1018^* (*n*=30) larvae were incubated in 0, 100 µM or 10 mM MTZ for 10 days (3 dpf to 13 dpf). Survival was plotted against time (Fig. 6A), and β cells were detected by fluorescence microscopy (Fig. 6B-D). Survival of larvae maintained without MTZ was ∼75% (regardless of whether NTR was present). Incubation in 100 µM MTZ had no effect on this level of survival, even with NTR-dependent loss of β cells (Fig. 6D). However, all larvae incubated in 10 mM MTZ died within the first 5 days of treatment. Altogether, these results indicate that (1) the NTR2.0 transgene has no effect on survival, (2) larval fish can be maintained for 10 days without β cells without effects on survival, and (3) any models requiring 10 mM MTZ incubation will be incompatible with modeling chronic hyperglycemia during development.

### Modeling chronic diabetes in adult zebrafish

**Fig. 7.**
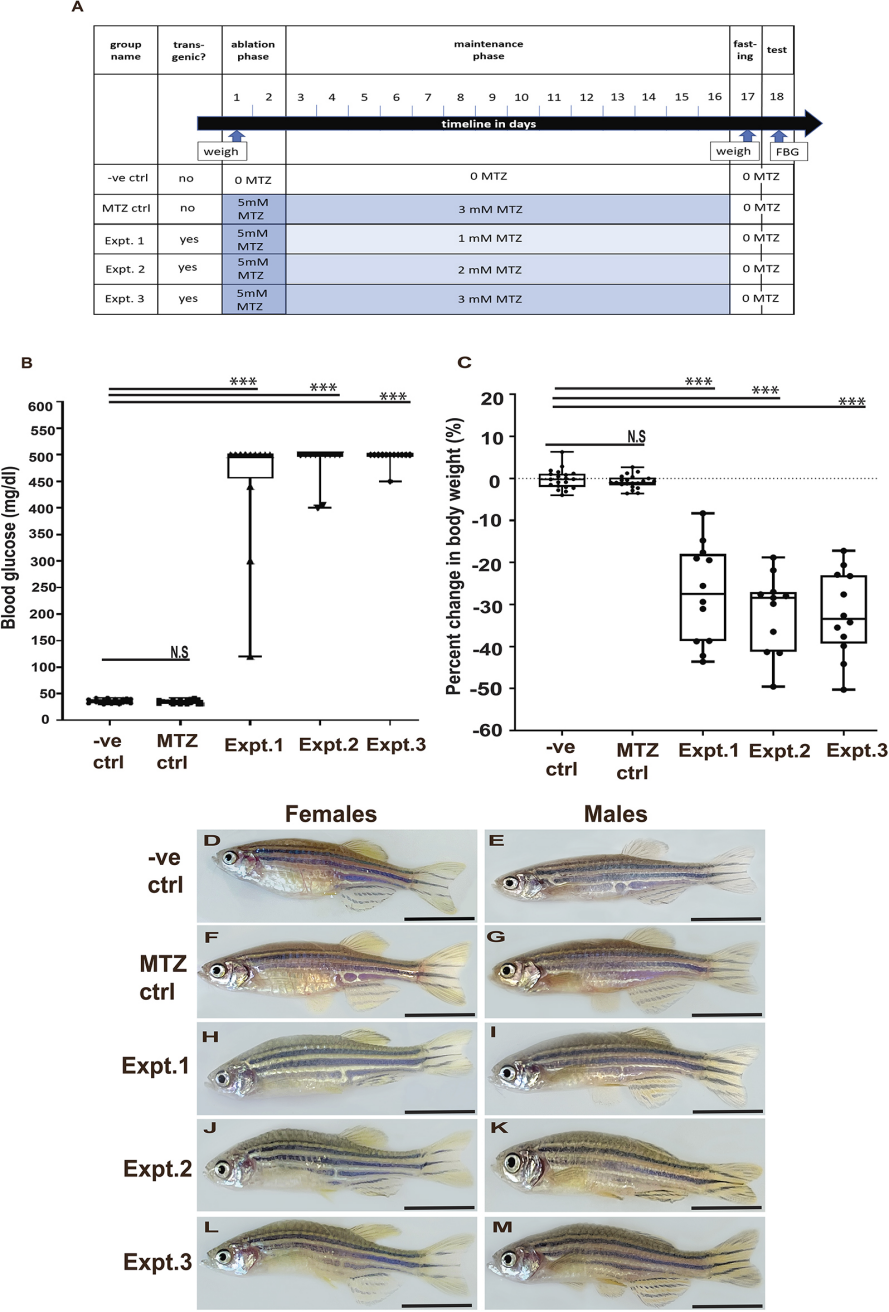
**Chronic hyperglycemia can be achieved in adults using NTR2.0 fish.** (A) Timeline of the chronic MTZ treatment trial. (B) Blood glucose readings of individual adult zebrafish from the following groups: negative controls (wild type, no MTZ, -ve. ctrl), MTZ controls (wild type with MTZ, MTZ ctrl) and three experimental groups (*ins:mCherry NTR2.0^ir2018^* fish treated with MTZ as outlined in A, Expt. 1-3). All fish in the experimental groups became hyperglycemic. Owing to the limitation of the blood glucose meter used, 500 mg/dl is the maximum blood glucose reading that can be captured. (C) Percentage change in body weight over the course of the trial for individual fish in control and experimental groups. The dotted line represents no change in body weight. (D-M) Representative images of female and male fish from control and experimental groups. Scale bars: 1 cm. N.S., nonsignificant; ***P<0.05 (unpaired two-tailed Student's *t*-test). FBG, fasting blood glucose; MTZ, metronidazole.

To test whether *ins:mCherry NTR2.0^ir2018^* adult fish can be used as a model to study chronic hyperglycemia, we performed the 18 day MTZ treatment trial outlined in Fig. 7A. All fish were weighed at the beginning (day 1) and towards the end (day 17) of the trial to detect potential changes in body weight. After this second weighing, all fish were photographed and then fasted for 24 h. On day 18, the fish were euthanized and FBG measurements were taken. We included two controls and three experimental groups. A negative control group (abbreviated to ‘-ve. ctrl’) consisted of wild-type fish (*n*=20) treated in an identical manner to the experimental groups but without any MTZ. A second control group accounting for potential NTR-independent effects of MTZ (abbreviated to ‘MTZ ctrl’) consisted of wild-type fish (i.e. no NTR2.0, *n*=20) maintained in the highest doses of MTZ being tested in the trial. Three experimental groups of *ins:mCherry NTR2.0^ir2018^* fish (abbreviated to ‘expt. 1’, ‘expt. 2’ and ‘expt. 3’) were initially immersed in 5 mM MTZ for 2 days in an ‘ablation phase’ to remove all β cells. An ablation ‘maintenance phase’ (days 3 to 16) was designed to stop the reappearance of new β cells and maintain hyperglycemia. For these 14 days, fish were kept in 1 mM MTZ (expt.1, *n*=12), 2 mM MTZ (expt. 2, *n*=11) or 3 mM MTZ (expt. 3, *n*=12). By the end of the trial, fish in the control groups (-ve. ctrl and MTZ ctrl) had similar mean (±s.d.) FBG levels of 35.9±3.94 mg/dl and 34.8±3.67 mg/dl, respectively (Fig. 7B), had maintained their body weight (Fig. 7C) and appeared healthy (Fig. 7D-G).

In contrast to controls, fish in all three experimental groups (expt. 1, expt. 2 and expt. 3) demonstrated hyperglycemia, with most fish showing an FBG measurement of ≥500 mg/dl (Fig. 6B). These fish also lost considerable body weight during the trial (Fig. 7C), a response independent of sex. Expt. 1 fish had a mean (±s.d.) FBG of 446.7±113 mg/dl, with 9/12 reaching ≥500 mg/dl, and had an average weight loss of 27±11.2%. Expt. 2 fish had an average FBG of 482.3±37.6 mg/dl, with 9/11 reaching ≥500 mg/dl and 31.8±8.83% average weight loss. Expt. 3 fish an average FBG of 495.8±13.8 mg/dl, with 11/12 reaching ≥500 mg/dl and 32.2±9.66% average weight loss. We expect that experimental fish with FBG levels lower than 500 mg/dl represent fish in which β-cell ablation was incomplete. It was noted that, by the second week of the maintenance phase of chronic hyperglycemia, experimental fish displayed anorexia. By the end of treatment, fish in all experimental groups and of both sexes appeared less colorful and possessed abnormally curved body shapes consistent with severe weight loss (Fig. 7H-M). Together, our results demonstrated that treatment for 2 days in 5 mM MTZ followed by 1 mM, 2 mM or 3 mM MTZ will keep NTR2.0 fish hyperglycemic for at least 16 days. We also observed significant NTR/MTZ-dependent weight loss during the experiment, consistent with pathological effects of a prolonged diabetic state.

## DISCUSSION

We describe two new transgenic tools that allow temporally controlled induction of chronic hyperglycemia in zebrafish. The *ins:mCherry NTR2.0^ir2018^* and *ins:YFP NTR2.0^ir2050^* fish are a new model system to study diabetes-associated pathology. The higher activity of NTR2.0 means that less MTZ is required for effective β-cell ablation, reducing potential issues of toxicity and gut inflammation. In the absence of MTZ, expression of NTR2.0 has no deleterious effects on larval or adult health, although we did observe MTZ-independent loss of β cells in an initial transgenic line and suspect that overexpression of membrane-targeted tagYFP is cytotoxic to β cells. Previous NTR1.0-based models required 10 mM of the antibiotic MTZ to induce cell-specific ablation. Here, we report that 10 mM is the LD_50_ for a 48 h MTZ treatment in both larval and adult zebrafish, and that surviving fish display severe damage to the intestinal epithelium. Consistent with our results, others have shown that *Onchorhynchus mykiss* (rainbow trout) also displays aberrant intestinal pathology when treated with MTZ (Gürcü et al., 2016). The mechanism of tissue damage in both fish is unknown, but we found evidence that in larval zebrafish the adverse effects of 10 mM MTZ are most likely independent of effects on residential microbial communities. Therefore, we concluded that 10 mM MTZ has direct toxicity in some larval tissues. In this study, we did not establish the cause of MTZ-induced pathology in adults. As the microbiome is known to change significantly from larval to adult stages (Stephens et al., 2016), it is possible that high doses of MTZ are deleterious to adult zebrafish via microbiome disruption, direct toxicity or a combination of both. Indeed, Morgun et al. (2015) showed that a cocktail of antibiotics (including MTZ) given to adult mice caused intestinal transcriptome (and physiological) changes via both direct effects and changes to the microbiome. Elucidating the mechanism of MTZ-induced pathology in adult zebrafish will require further experimentation.

With improved NTR2.0 ablation efficacy, we were able to simplify drug delivery. Previously, NTR1.0 adult fish had to be injected intracelomically with a single dose of 10 mM MTZ to effectively ablate β cells (Delaspre et al., 2015; Huang et al., 2016). Injecting fish is technically challenging as the fish are relatively small. Injections can result in accidental death and, if not administered carefully, can cause some β cells to escape ablation. With *ins:mCherry NTR2.0^ir2018^* fish, acute β-cell ablation can be induced by simple immersion in 10 mM MTZ for 24 h or 5 mM MTZ for 48 h. Although both treatments induced the maximum hyperglycemia we can measure (FBG of 500 mg/dl), fish treated with 5 mM MTZ for 48 h were healthier than those treated with 10 mM MTZ, with no evidence of intestinal damage. It is very likely that lower concentrations of MTZ for longer periods over 48 h would also induce complete β-cell ablation.

In summary, we conclude that *ins:mCherry NTR2.0^ir2018^* and *ins:YFP NTR2.0^ir2050^* fish are appropriate tools for modeling the complications associated with hyperglycemia such as neuropathy, nephropathy, retinopathy, diabetic ketoacidosis and arterial disease. However, depending on experimental goals, the following should be carefully considered: (1) what age is appropriate for hyperglycemia induction, (2) how long a period of hyperglycemia is required, and (3) what level of hyperglycemia is most relevant to the study planned.

(1) Age of induction: in considering models of chronic hyperglycemia, the main advantage of transgenic methods over genetic mutants, is the opportunity to incorporate inducibility. The ability to temporally control when β cells are ablated allows the effects of chronic hyperglycemia on adult physiology to be separated from developmental effects. As we have shown, chronic hyperglycemia can be maintained in larval development, providing a potential model for gestational diabetes, or hyperglycemia can be induced post-larval stages, to resemble the onsets of T1D and T2D more closely.

(2) Duration of hyperglycemia: in designing our chronic hyperglycemic studies, we rationalized that 48 h immersion in 5 mM MTZ would ablate all β cells (as per our acute method) and then a lower dose would maintain hyperglycemia. The use of a lower dose was a compromise between preventing β-cell regeneration and keeping fish healthy. During the first week of the maintenance phase, fish in the experimental groups (*ins:mCherry NTR2.0^ir2018^* fish in MTZ) showed few visible changes in their overall appearance and behavior. However, by the second week, these fish displayed a rapid decline in health, loss of appetite and visible weight loss. Continuing our experimental conditions beyond 14 days would likely lead to lethality. We believe that it would be possible to extend the duration of hyperglycemia by modifications to the MTZ dosing regimen to modulate the level of induced hyperglycemia.

(3) Levels of hyperglycemia: in our trial to induce chronic hyperglycemia, we had three experimental groups (expt. 1, expt. 2 and expt. 3), and in all groups a hyperglycemic state was achieved. Fish in expt. 3 (treated with 3 mM MTZ) displayed the most extreme hyperglycemic outcome but also appeared the sickest – more lethargic than other fish and harder to exsanguinate for FBG measurements. If the goal of future studies is to study complications associated with diabetes, going to this extreme of hyperglycemia may not be necessary or suitable. Fish in expt. 1 (treated with 1 mM MTZ) were all hyperglycemic but showed greater variation in FBG. These results are consistent with regenerated β cells providing some insulin in some fish (3/12) to influence their FBG levels. We rationalize that maintenance doses of 1-2 mM MTZ (or lower) should be sufficient to elicit enough hyperglycemia to model the pathology seen in hyperglycemic patients (i.e. visible weight loss and lack of activity) (Roche et al., 2005).

In humans, there is accumulating evidence that the occurrence and development of diabetic complications are not just related to constant levels of extreme high glucose concentrations but are often caused by glucose fluctuations (Zhang et al., 2019). Accordingly, a method to extend hyperglycemia and match physiology in diabetic patients would be to carry out cycles of ablation and recovery, for instance placing fish in MTZ for 2 days, allowing regeneration for 5 or more days and repeating.

Besides modeling hyperglycemia-associated pathophysiology, we believe that another utility of *ins:mCherry NTR2.0^ir2018^* and *ins:YFP NTR2.0^ir2050^* fish could be further characterization of β-cell regeneration. Studies using acute β-cell ablation in adult zebrafish have identified several cellular origins of regenerated β cells, including ductal associated centroacinar cells (Delaspre et al., 2015; Ghaye et al., 2015), as well as islet-localized α cells (Ye et al., 2015), δ cells (Carril Pardo et al., 2022) and ε cells (Yu et al., 2023). With the new ability to cause continuous β-cell ablation, we can expand this work and ask questions about these cellular origins. Can these sources be depleted? Are these sources replenished and, if so, how? And are there other unknown origins of new β cells still to be discovered?

In conclusion, our novel NTR2.0 expressing lines will allow those in the field to interrogate β-cell regenerative potential after acute or chronic β-cell loss, and it will also help to expand chronic hyperglycemia disease modeling at pathological and molecular levels.

## MATERIALS AND METHODS

### Zebrafish lines

*ins:mCherry NTR2.0*^ir2018^ and *ins:YFP NTR2.0*^ir2050^ transgenic fish were made using the transgenes *ins:mCherry-P2A-nfsB_Vv-F70A/F108Y* and *ins:YFP-P2A-nfsB_Vv-F70A/F108Y*. A *YFP-P2A-nfsB_Vv-F70A/F108Y* cassette from the construct *5xUAS:GAP-tagYFP-P2A-nfsB_Vv-F70A/F108Y* (Sharrock et al., 2022) was cloned downstream of the insulin promoter in a Tol2 transposable element (Pisharath et al., 2007). The YFP gene was removed and swapped for mCherry to create *ins:mCherry-P2A-nfsB_Vv-F70A/F108Y*. Eight NFκB-binding elements and the CMV minimal promoter [from the plasmid pSGNluc NFκB reporter (Kuri et al., 2017)] were cloned upstream of GFP in a Tol2 transposable element to create *8xNFκB:GFP*, the construct injected to generate *8xNFκB:GFP^ir2019^* fish. All DNA constructs mentioned above were mixed with Tol2 transposase mRNA and Phenol Red (as per Kawakami, 2004) and injected into single-cell embryos to generate F0 mosaic fish. These F0s were raised and outcrossed to generate F1 founders. These F1 fish were screened by outcrossing to wild-type fish, followed by observation of F2 progeny to ensure the expected expression pattern. Transgenic F2 fish were raised and outcrossed again to identify F2 fish transmitting the transgene at 50% – i.e. to isolate F2 hemizygotes with a single insertion of the transgene. *casper* fish lack pigment in the body (White et al., 2008) and are homozygous mutant for *mitfa* (Lister et al., 1999) and *mpv17* (D'Agati et al., 2017). Zebrafish experimental protocols were approved by and performed in accordance with the Institutional Animal Care and Use Committee at University of California, Irvine. In an effort to facilitate distribution, transgenic fish have been offered to the Zebrafish International Resource Center.

### Drug-dependent cell ablation

In all acute and chronic larvae/adult experiments, the water/MTZ solution containing the fish was completely changed daily. MTZ (AC210341000, Thermo Fisher Scientific) solutions were made fresh (necessary for optimal function). It is important to note that other commercial sources of MTZ tested had higher batch variability in terms of ablation efficacy and toxicity. Larvae (3-5 dpf, *n*=25-50) were kept in MTZ dissolved in E3 medium (Westerfield, 1995) in Petri dishes at 28°C. Individual adults were kept in system water in 200 ml beakers at 28°C. Adult fish were fed daily (Gemma 500, Skretting). For chronic β-cell ablation studies in larvae, 3-5 dpf fish were treated as above. At 6 dpf larvae (*n*=20) were transferred to small beakers and fed once per day (Gemma micro 75, Skretting) before MTZ-treated system water was 100% exchanged. For chronic studies in adults, individual fish were kept in 1 l tanks containing 250 ml MTZ dissolved in system water. Adults were fed 0.077 g Gemma 500 once a day and given 3 min to eat before an MTZ/water exchange was performed. Feeding of live food was omitted to prevent potential MTZ metabolism outside of the fish.

### H&E staining of paraffin sections

For all groups, fish were given an ID number, and corresponding treatment information associated with each fish was kept in a separate document. Adult fish were euthanized on ice. A small incision along the midline of the fish was made before placing each cadaver in 10% non-buffered formaldehyde and leaving overnight at room temperature. For adult gut histology, cadavers can be fixed indefinitely, but for pancreas studies fixation was limited to overnight. After fixation, guts/pancreas were removed to 70% ethanol for 1 h. Tissue was placed into cassettes (62500 series, Tissue Tek), processed in a tissue processor (TP1020, Leica) overnight and embedded in paraffin the next day (Sabaliauskas et al., 2006). Sagittal sections (5 µm) were cut on a microtome and stained with H&E. For all control and treatment groups in adults, we looked at the gut histology of five fish for each group, which surmounted to 25 histological slides per fish. Each histological slide contained four to five sections of sectioned gut/pancreas tissue. When looking at H&E staining of the gut, fish were scored blindly for gut abnormalities and then their corresponding ID number was referenced back to a document containing information about their treatment in order to draw conclusions for each group.

For larvae, the head of the fish was cut off after overnight fixation in 10% non-buffered formaldehyde. Larvae were processed similarly to the adult fish mentioned above. Transverse sections (5 µm) were cut on a microtome. We looked at the gut histology of five fish for each group. We obtained three to four slides per fish, with each slide containing three to four sections of gut tissue. All slides were H&E stained to establish the effect of MTZ the gut of larval fish. Scoring the larval guts was done the same as in adult fish.

### Immunofluorescence

Immunofluorescence staining was performed on adult sections of guts containing pancreas (Parsons et al., 2009). For deparaffinization/rehydration steps, sections on microscope slides were placed in a slide rack and fully submerged in solutions. Histo-Clear (National Diagnostics) was used as a substitute for xylene. Paraffin sections underwent antigen retrieval using standard procedures with citrate (as per the supplier's protocol, Cell Signaling Technology). Slides were then fully submerged in 1× antigen retrieval buffer (100× citrate buffer; ab93678, Abcam) at room temperature for 10 min. While still in the slide rack, slides were transferred to a histology container containing boiling deionized water. The histology container contained pre-warmed 1× antigen retrieval buffer, and slides were incubated in this buffer for 10 min. The antigen retrieval container with the slides was removed and cooled on ice for 30 min. Slides were then removed from the slide rack, dried carefully with a Kimwipe™ and then placed in an incubation chamber containing deionized water. A PAP pen was used to draw a circle around each individual tissue on each slide. Tissue sections were then incubated in ∼15-20 µl permeabilization buffer (0.5% Triton X-100, 1× PBS) for 20 min to increase primary antibody penetration. Tissue sections were then incubated in 15-20 µl freshly made block buffer (10% goat serum, 0.2% Triton X-100, 1× PBS) at room temperature for 1 h.

The primary antibodies used [1:200 rabbit anti-insulin (GTX101136, Genetex), 1:200 mouse anti-glucagon (G265, Sigma-Aldrich) and 1:200 rat anti-mCherry (M11217, Invitrogen)] were diluted in fresh block buffer and incubated overnight at 4°C. The next day, the primary antibody was pipetted off each section and slides were washed completely with wash buffer (0.2% Triton X-100, 1× PBS) 3× for 5 min. Slides were dried carefully with a Kimwipe™, tissue samples were circled again with a PAP pen, and 10-15 µl freshly made block/wash buffer (10% goat serum, 0.2% Triton X-100, 1× PBS) was added for a 5 min wash and then pipetted off. Each tissue sample was then incubated for 2 h in block/wash buffer containing 1:2000 4′,6-diamidino-2-phenylindole (DAPI; D9542, Sigma-Aldrich), 1:250 anti-rabbit Alexa Fluor 488 (ab150077, Abcam), 1:250 anti-mouse Alexa Fluor 594 (ab150116, Abcam) and 1:250 anti-rat Alexa Fluor 594 (ab150160, Abcam) secondary antibodies. After, slides were washed completely with wash buffer followed by a 2 min wash with 1× PBS. Slides were then mounted with a cover slip for imaging.

### FBG assays

Adult fish were fasted for 24 h in breeding tanks to prevent coprophagia, before being euthanized on ice. Fish were then decapitated, and a test strip was used to draw up blood to a FreeStyle lite (Abbott) glucose meter. It should be noted that 500 mg/dl is the maximum blood glucose level this meter can detect, and it is known that the hyperglycemia in zebrafish can be considerably higher (Delaspre et al., 2015).

### Image acquisition

All fluorescent images were acquired using a Leica SP8 confocal microscope. Live embryos at 5 dpf were anesthetized with tricaine and mounted in 1% low-melt agarose for imaging. H&E images were acquired using an EVOS XL Core microscope.

### PCR analysis

Touchdown PCR was performed using PCRBIO Taq Mix Red (PCR Biosystems): 95°C for 4 min, followed by ten cycles of 95°C for 30 s, 69°C for 30 s and 72°C for 30 s. Each progressive cycle's annealing temperature dropped by 1°C. This was followed by 27 cycles of 95°C for 30 s, 59°C for 30 s and 72°C for 30 s and then 72°C for 4 min. Sequences of primers shown in Fig. S3 are as follows: F1, 5′-TGTTCCACACAGGTCAGAGG-3′; R1, 5′-TTGAGTAGCGTGTACTGGCATT-3′; F2, 5′-CACCATCGTGGAACAGTACG-3′; R2, 5′-CAACGTGAGAAGCATCCAAA-3′; F3, 5′-GGTTCTTGACCCCCTACCTT-3′; R3, 5′-ATTAATGCAGCTGGCACGAC-3′; F4, 5′-CCGACCACTACCAGACCAAC-3′; and R4, 5′-CACTGCTCGCGACAATAAAA-3′.

### Germ-free derivation

*8xNFκB:eGFP* embryos were derived GF as previously described (Melancon et al., 2017) with slight modification. Briefly, fertilized eggs from adult mating pairs were collected and incubated in sterile embryo medium (EM) containing ampicillin (100 µg/ml; Gold Biotechnology), gentamicin (10 µg/ml; Gold Biotechnology), amphotericin B (250 ng/ml; Gold Biotechnology), tetracycline (1 µg/ml; Gold Biotechnology) and chloramphenicol (1 µg/ml; Gold Biotechnology) for ∼5 h. Embryos were then washed in EM containing 0.1% polyvinylpyrrolidone-iodine followed by EM containing 0.003% sodium hypochlorite. Surface-sterilized embryos were distributed into sterile T25 and T75 tissue culture flasks at a density of one embryo/ml. CVZ embryos were generated by inoculating flasks containing GF embryos with undefined microbial communities derived from parental zebrafish feces immediately following GF embryo derivation. Zebrafish embryos and larvae were kept at 28.5°C in an incubator with a 14 h/10 h light/dark cycle.

For MTZ treatments, EM containing 10 mM MTZ was made fresh daily and filter sterilized using a vacuum-driven filter (25-233, Genclone). Sterile technique was used to replace the original EM in flasks with EM containing MTZ. MTZ-containing EM exchanges were performed on 3 and 4 dpf followed by assessment of GF status using an inverted microscope to inspect flask water for microbial contaminants. Confocal imaging was carried out on 5 dpf to inspect transgene activation.

### Statistical analysis

Significance of blood glucose and body weight results were assessed using unpaired two-tailed Student's *t*-test in GraphPad Prism. *P*<0.05 was considered significant. s.d. was calculated for both blood glucose and body weight changes to measure the variation of the data in relation to the mean.

## Supplementary Material

10.1242/dmm.050215_sup1 Supplementary information
