# Supplementary material for: An inducible model of chronic hyperglycemia
Source: Dis Model Mech. 2023 Aug 4;16(8):dmm050215. doi: 10.1242/dmm.050215 (PMC10417516; doi:10.1242/dmm.050215)
Supplement: Supplementary information [file dmm-16-050215-s1.pdf]

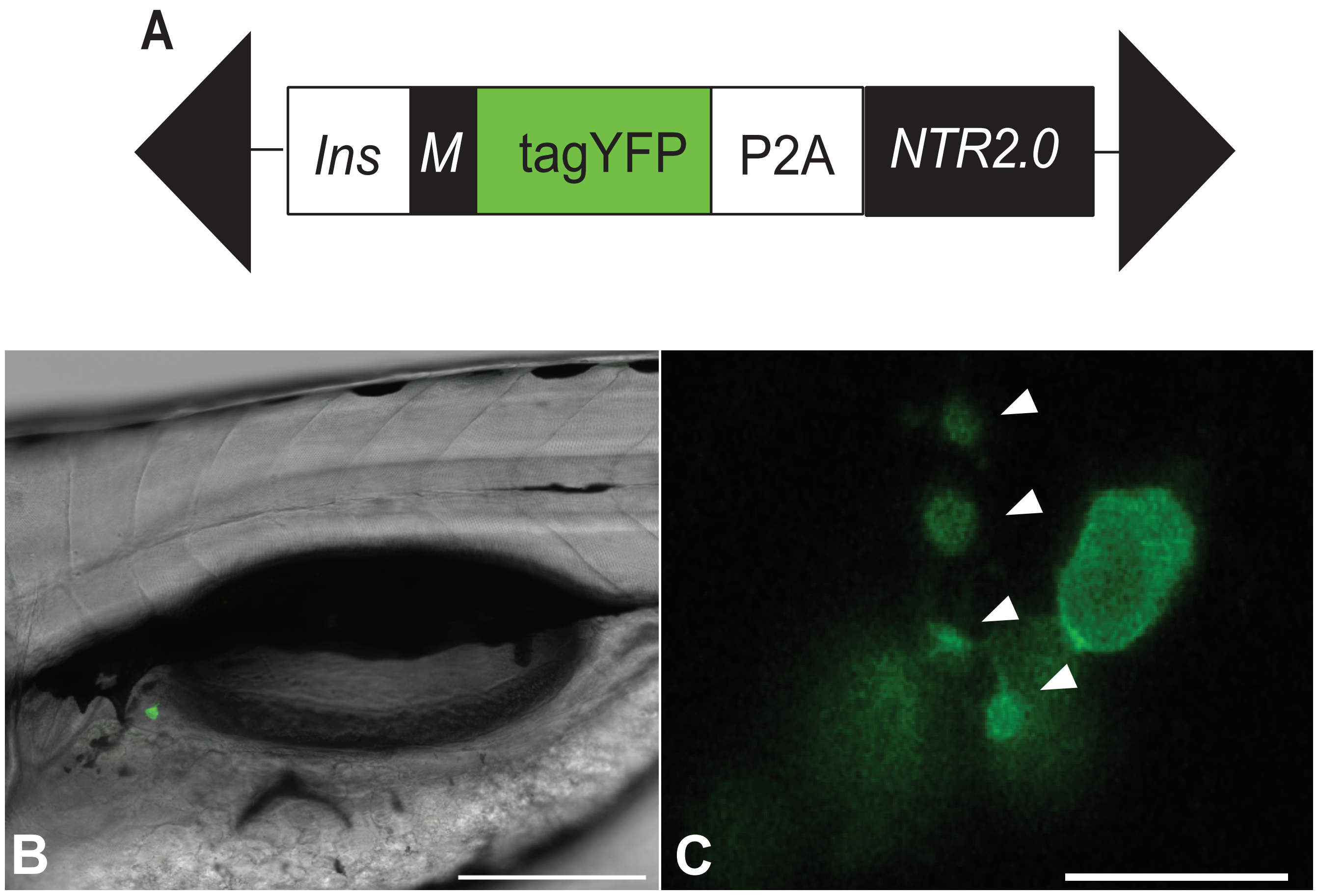

Fig. S1. Membrane expressing NTR2.0 YFP transgenic fish

(**A**) Construct used to generate transmembrane NTR2.0 YFP transgenic fish, *ins:tagYFP-P2A-NTR2.0*. Tol2 arms (black triangles), the insulin promoter (box - ins), the palmitoylation sequence from GAP43 (box- M) that confers membrane localization to the fluorescent protein (tag YFP) and the gene that encodes NTR2.0 (box-NTR2.0) (M) (**B**) Low magnification image of fish via confocal microscopy showing only a single cell fluorescence in the position of the pancreas (**C**). Higher magnification image of fish via confocal microscopy, showing the cells expressing transmembrane YFP are blebbing (white arrows) consistent with cell stress. Concluded that high expression of membrane tagged YFP is toxic to the β cells.

Disease Models & Mechanisms • Supplementary information

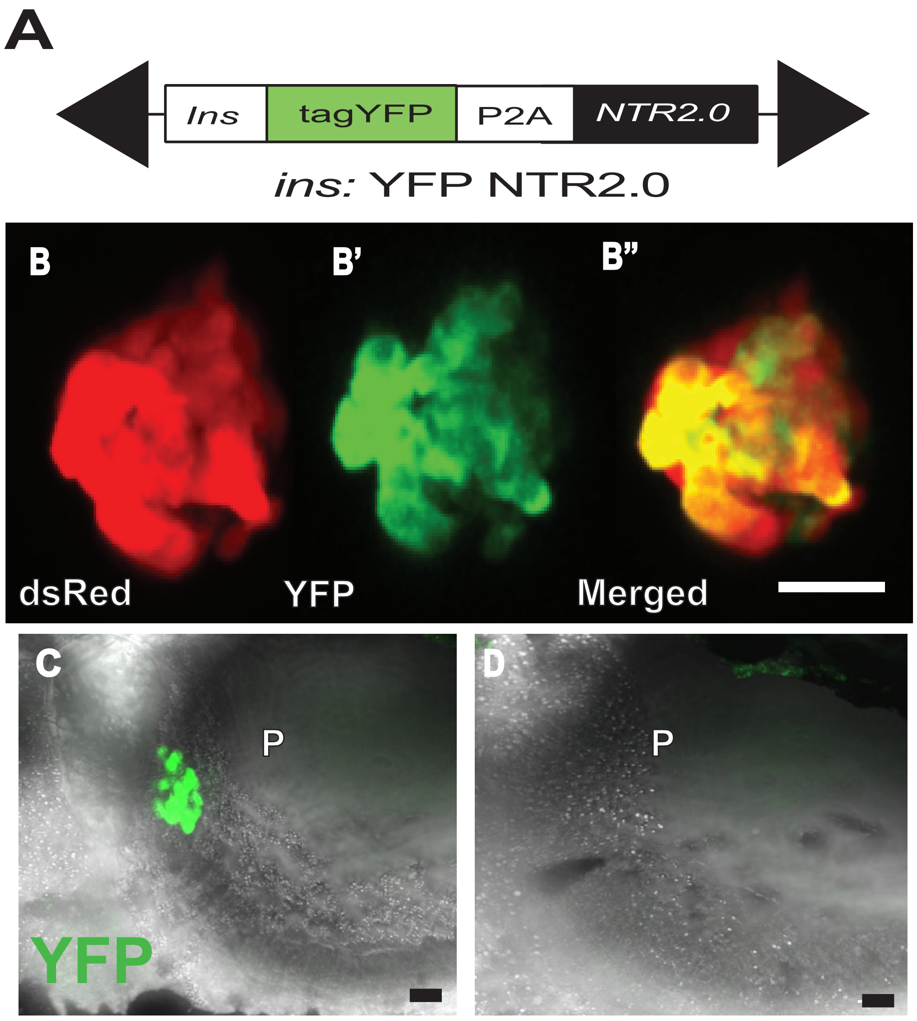

Fig. S2. *ins:YFP NTR2.0* transgenic line

(**A**) We created a YFP version of NTR2.0 to label and ablate β cells. Tol2 arms (black triangles), the insulin promoter (box - ins), and sequences encoding the yellow fluorescent protein (tagYFP) and the gene that encodes NTR2.0 (box-NTR2.0). The P2A sequence ensures YFP and NTR2.0 are produced as separate proteins. Live imaging was performed on 5 dpf larvae (n=20) expressing a transgene known to mark the cytoplasm of all β cells (*ins:dsRed*) and tagYFP (YFP) (**B-D**). (**B-B''**) Demonstrates that tagYFP labels all β cell in 5 dpf larvae. (**C**) Represents tagYFP in larvae without MTZ treatment. (**D**) Representative larvae (n=20) treated with 100mM MTZ from 3dpf to 5dpf - demonstrates that expression of tagYFP and NTR2.0 leads to complete β-cell ablation. Images were taken at 40× (scale bar = 20 μM). P marks the position of the head of the pancreas where the islet should be located.

Disease Models & Mechanisms • Supplementary information

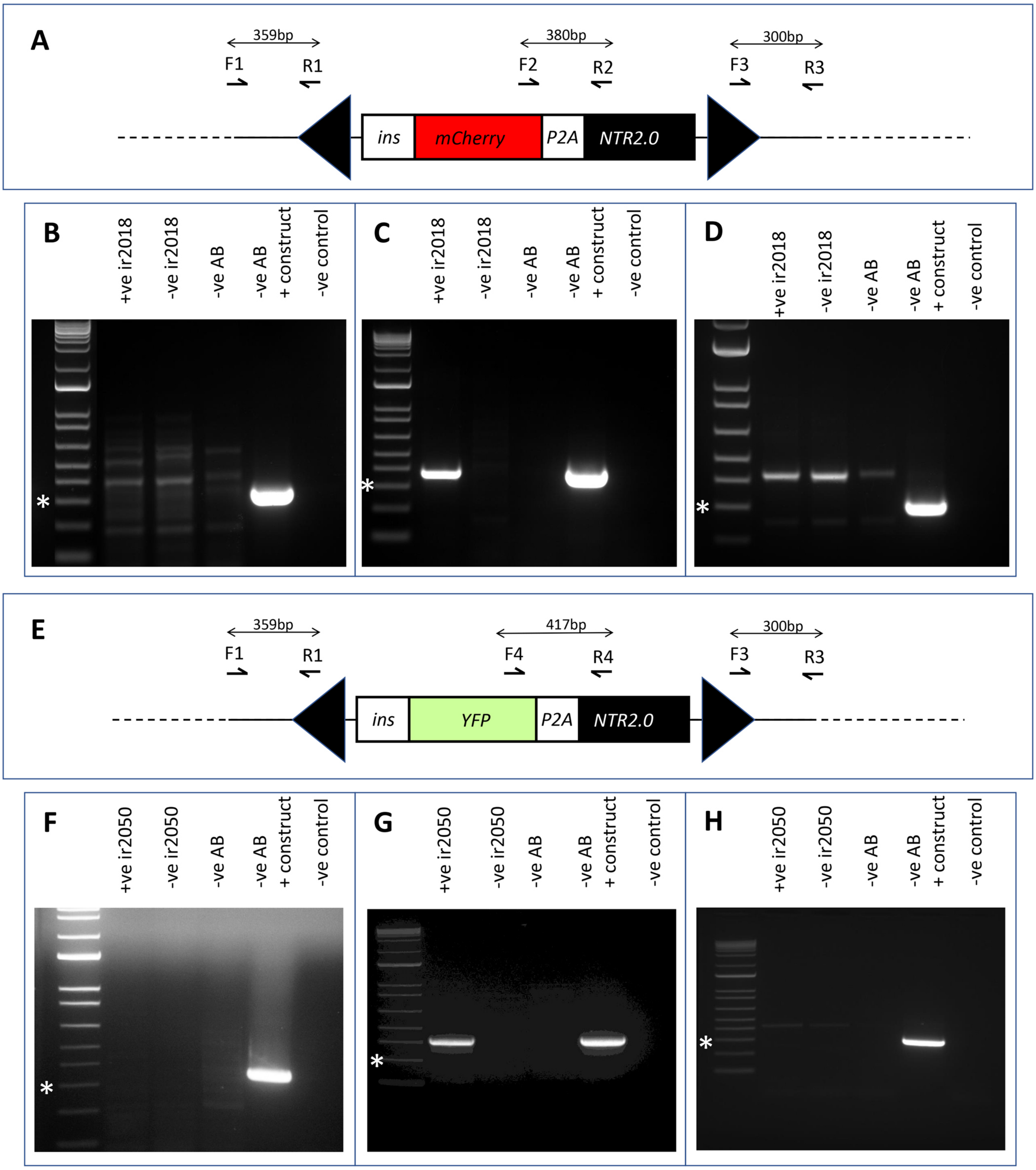

Disease Models & Mechanisms • Supplementary information

Fig. S3. Transgenes integrated via Tol2-mediated transgenesis

(**A** and **E**) Schematics of the constructs used to generate the fish lines *ins:mCherry NTR2.0*^{ir2018} and *ins:YFP NTR2.0*^{ir2050}. Black triangles represent Tol2 arms. Position of insulin promoter is shown (white box, ins). Genes encoding mCherry (red), tagYFP (green), the P2A self-cleaving peptide (white, P2A) and NTR2.0 (black). The position of primers used in PCRs are also indicated (half arrows), as are the sizes of PCR products (double-headed arrows). Results of PCRs using primers: F1 and R1 in (**B)**, F2 and R2 in (**C**)**,** F3 and R3 in (**D**), F1 and R1 in (**F)**, F4 and R4 in (**C**)**,** F3 and R3 in (**H**). (**B-D**) Examination of genomic integration of *ins:mCherry NTR2.0*^{ir2018} 1st lane DNA template was extracted from a fluorescent positive *ins:mCherry NTR2.0*^{ir2018} larvae (+ve ir2018). 2nd lane from a fluorescent negative sibling (-ve ir2018). 3rd lane from a wildtype fish (-ve AB). 4th lane from same wildtype fish spiked with 80pg of construct shown in (**A**) (-ve AB + construct). 5th lane template was lysis buffer that went through whole DNA extraction protocol with other samples (-ve control). (**F-H**) Same set up but using DNA from *ins:YFP NTR2.0*^{ir2050} larvae and the construct shown in (**E**). DNA ladder is 1kb+ from Invitrogen and the 300bp band is marked by a *. Band above is 400 and the band below is 200.

PCRs using primers internal to the Tol2 arms only generate amplicons when fluorescent fish is the template or when construct DNA is added. PCRs using primers external to the Tol2 arms only generate amplicons when construct DNA is added. Together, this demonstrates that both *ins:mCherry NTR2.0*^{ir2018} and *ins:YFP NTR2.0*^{ir2050} fish are transgenic curtesy of Tol2-mediated transposition. Non-specific amplification can be seen in several PCRs, but these products are weak, not the predicted size, occur in both wildtype and negative sibling DNA and, most importantly, are abolished by the addition of the construct (4th lanes).

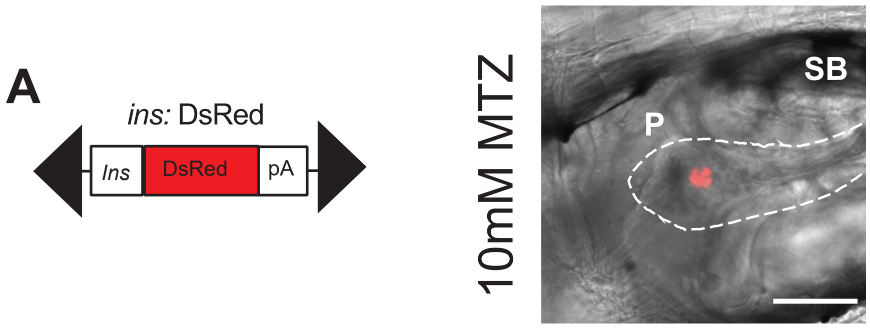

Fig. S4. Without NTR, MTZ does not lead to β-cell death

(**A**) Schematic of the ins:DsRed transgene. Black triangles represent Tol2 arms. Position of insulin promoter is shown (white box, ins). Genes encoding dsRed (red) (**B**) Larva (5 dpf) expressing ins:DsRed after treatment with 10mM metronidazole (3-5 dpf) to demonstrate that prodrug does not kill β cells in absence of NTR expression (n =30). Image was taken at 40× (scale bar = 100 μM).

Disease Models & Mechanisms • Supplementary information

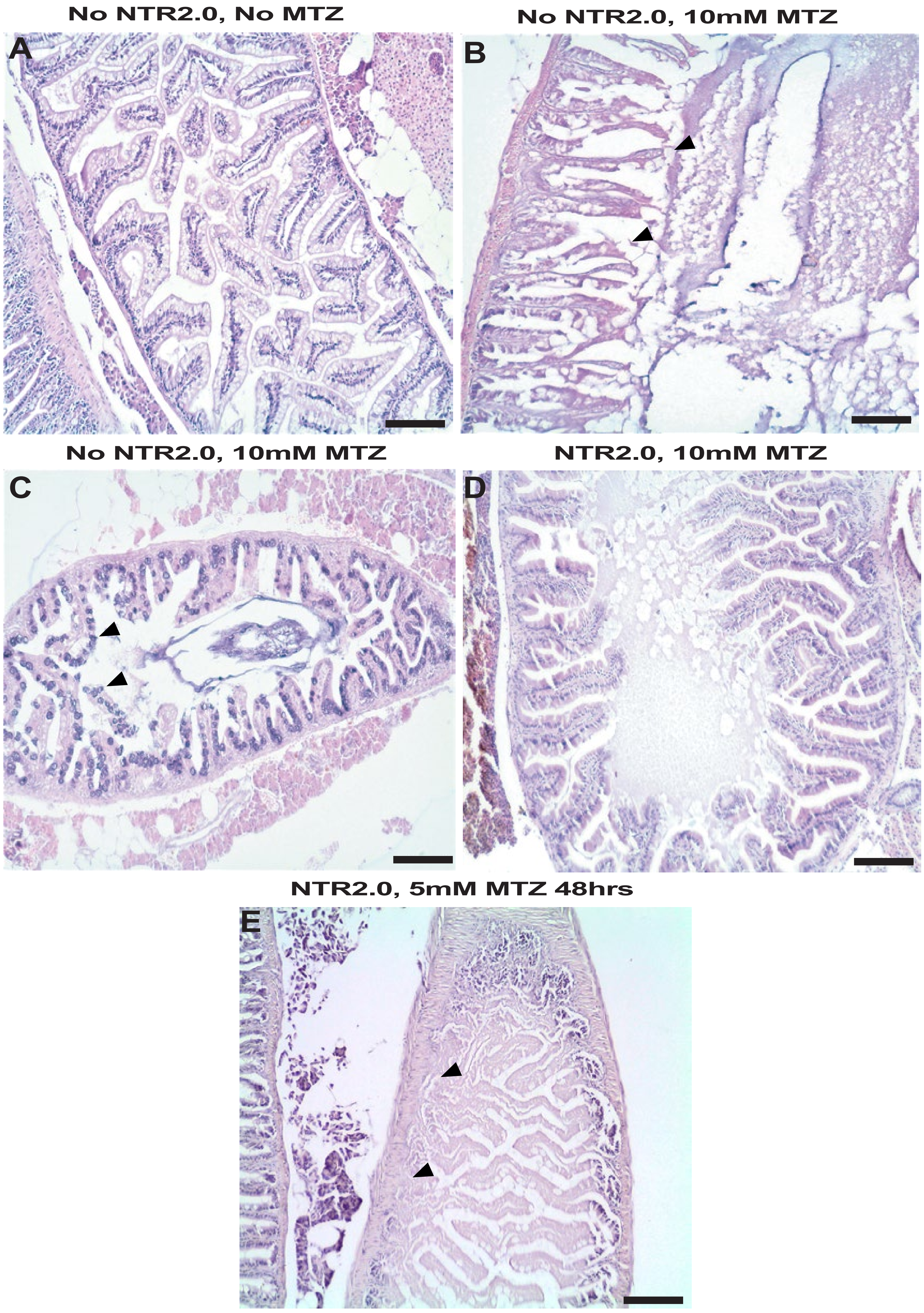

Disease Models & Mechanisms • Supplementary information

Fig. S5. Histological analysis of the gut in fish treated with MTZ

H & E staining on adult gut sections of fish treated with and without MTZ. (**A**) Region of the gut in wildtype fish that were not treated with MTZ (20×, scale bar = 100 μM). (**B**) Region of the gut where the lining has been damaged (black arrows) in wildtype fish immersed for in 10mM MTZ for 24 hrs (20×, scale bar = 100 μM). This damage was consistently seen in all 5 fish looked at for this group. (**C**) Is a region of the gut where one out of the 5 fish in this group (wildtype fish immersed for 24 hrs in 10mM MTZ) had an increase in the number of goblet cells present (black arrows) (20×, scale bar = 100 μM). (**D**) Is a region of the gut that appeared normal in NTR2.0 fish that were immersed in 10mM MTZ for 24 hrs. In all 5 fish looked at for both wildtype and NTR2.0 fish treated with 10mM MTZ for 24 hrs, there was consistent damage as seen in (**B**) interspersed throughout normal gut tissue as seen in (**D**). (**E**) One out of the 5 fish treated (NTR2.0 fish immersed for 48 hrs in 5mM MTZ) had a single region of the gut with damage to the intestinal lining (black arrows) (20×, scale bar = 100 μM).

Disease Models & Mechanisms • Supplementary information
